# Evaluation of interactive web-based tools to stimulate reflection and communication about advance care planning with people with dementia and their family caregivers

**DOI:** 10.1186/s12904-024-01486-4

**Published:** 2024-06-28

**Authors:** Fanny Monnet, Lara Pivodic, Charlèss Dupont, Tinne Smets, Aline De Vleminck, Chantal Van Audenhove, Lieve Van den Block

**Affiliations:** 1 End-of-Life Care Research Group, Vrije Universiteit Brussel (VUB) & Ghent University, Laarbeeklaan 103, Brussels, 1090 Belgium; 2 Department of Family Medicine and Chronic Care, Vrije Universiteit Brussel (VUB), Brussel, Belgium; 3 LUCAS Center for Care Research and Consultancy, KU Leuven, Leuven, Belgium

**Keywords:** Advance care planning, Communication, Reflection, Web-based tools, Dementia

## Abstract

**Background:**

People with dementia and their family caregivers often encounter challenges in engaging in advance care planning (ACP), such as a lack of information and difficulties in engaging in ACP conversations. Using a user-centred design, we developed two interactive web-based tools as part of an ACP support website to stimulate ACP reflection and communication: (1) the ‘Thinking Now About Later’ tool, with open-ended questions about ‘what matters most’, and (2) a digital version of the ‘Life Wishes Cards’, a card tool with pre-formulated statements that prompt reflection about wishes for future care. This study aimed to evaluate the use of and experiences with two web-based tools by people with dementia and their family caregivers.

**Methods:**

During an eight-week period, people with dementia and family caregivers were invited to use the ACP support website in the way they preferred. The mixed-methods evaluation of the ACP tools involved capturing log data to assess website use and semi-structured qualitative interviews to capture experiences. Analyses included descriptive statistics of log data and framework analysis for qualitative data.

**Results:**

Of 52 participants, 21 people had dementia and 31 were family caregivers. The ‘Thinking Now About Later’ tool and ‘Life Wishes Cards’ were accessed 136 and 91 times respectively, with an average session duration of 14 minutes (SD = 27.45 minutes). 22 participants actively engaged with the tools, with the majority using the tools once, and seven revisiting them. Those who used the tools valued the guidance it provided for ACP conversations between people with dementia and their family caregivers. Participants reported that people with dementia experienced barriers to using the tools on their own, hence family caregivers usually facilitated the use and participation of people with dementia. Some highlighted not knowing what next steps to take after completing the tools online.

**Conclusions:**

Although less than half the people used the ACP tools, those who used them found them helpful to facilitate communication between people with dementia and their family. Family caregivers of people with dementia played a crucial role in facilitating the use of the web-based tools.

**Supplementary Information:**

The online version contains supplementary material available at 10.1186/s12904-024-01486-4.

## Introduction

Advance care planning (ACP) refers to a dynamic process in which individuals can explore and identify their values, reflect upon the meanings and consequences of serious illness scenarios, define their goals and preferences for future care, identify proxy decision makers, and document preferences [1, 2]. It is an ongoing communication process between patients, families, and health professionals [1, 2]. ACP often focuses on considerations related to end-of-life and medical care preferences. However, our previous research has shown that people with dementia and their family caregivers find it important to discuss ‘what matters most’ for the future, without solely focusing on end-of-life and medical preferences [3]. This perspective resonates with the public health approach to ACP which considers ACP as a way to normalise conversations about end-of-life preferences, death and dying, and a way to align medical and physical concerns with broader concerns of both patients and family caregivers [4–6].

ACP can be relevant and valuable for people with dementia and their family caregivers [7–9]. Considering the progressive cognitive decline associated with dementia, ACP provides people with dementia the opportunity to express preferences for future care [10]. Additionally, engaging in ACP may enable family caregivers to gain insight into the values and preferences of the person with dementia, as their role in the decision-making process becomes more important [11]. Nevertheless, people with dementia and family caregivers often encounter significant barriers to engaging in ACP, including challenges such as lack of information about what ACP is and difficulties in initiating and engaging in ACP conversations [10, 12].

Using a user-centred design, we developed an ACP support website for and with people with dementia and family caregivers [13] with the aim to inform people with dementia and family caregivers about ACP and support them in reflecting and communicating about ACP. The website incorporates information about what ACP is and its relevant legal framework in Belgium, provides communication support on how to start discussing ACP within families and with health professionals, and advises people with dementia and their family caregivers to discuss their wishes with healthcare professionals. To support ACP reflection and communication, the website includes two interactive ACP tools: the ‘Thinking Now About Later’ tool, which is a fill-in reflection and communication tool with open-ended questions about ‘what matters most to you’, developed specifically for this website, and the ‘Life Wishes Cards’ tool (Levenswensen kaarten in Dutch) with pre-formulated statements that prompt reflection about what is important for future care and treatment, based on the Go-Wish cards developed in the United States [14] and the cultural adaptation and translation of the Go-Wish cards in Belgium [15].

This study aimed to explore the use of the two web-based reflection and communication tools by people with dementia and family caregivers and to evaluate their experiences with using them.

## Methods

Over an 8-week period, people with dementia and their family caregivers were invited to use the ACP support website in the way they preferred. We used a mixed-method evaluation design, i.e. use was evaluated by capturing the log data (continuous data collection logging user activity on the website) and through semi-structured qualitative interviews to assess user experiences. This study was part of a larger evaluation study of the ACP support website, which is published separately [18].

### Overview of the web-based reflection and communication tools for ACP

Using a user-centred, evidence-based, and theory-informed design process, we have developed an ACP support website for and with people with dementia and their family caregivers in Flanders, the Dutch-speaking part of Belgium [13]. The website aims to inform and support people with dementia and their family caregivers in communicating and engaging in ACP.

The development process included people with dementia, family caregivers, healthcare professionals, and regional dementia associations in Flanders. As part of this ACP website, two web-based ACP tools were developed and tested, focused on stimulating reflection and communication concerning ACP between people with dementia and their family caregivers: (1) the ‘Thinking Now About Later’ tool, with open-ended questions about ‘what matters most’, and (2) a digital version of the ‘Life Wishes Cards’ (Levenswensen kaarten in Dutch), a card tool with pre-formulated statements that prompt reflection about what is important for future care, based on the Go Wish cards developed in the US [14, 15]. To increase user-friendliness, tutorial videos are provided to explain how to use each web-based tool. Print and save options are also offered, so that users can record their progress.

#### Tool 1: the ‘Thinking now about later’ web-based tool

The ‘Thinking Now About Later’ tool is a self-guided fill-in tool designed to facilitate a reflective process regarding ACP. Through prompts and questions, users are guided to contemplate and discuss their present and future preferences, with a focus on identifying ‘what matters most’.

The need for a focus on ‘what matters most’ became apparent as part of a qualitative study with the European Working Group of People with Dementia (i.e. a multinational group composed of people with dementia who are nominated by their national associations, and their supporters, coordinated by Alzheimer Europe), which we had performed earlier to inform the development process of the ACP support website. This work highlighted the need for strengthening the focus on social aspects of care in ACP and on what matters most to people for their future. The European Working Group of People with Dementia found that current ACP definitions focus too much on medical care alone, and recommended that broader aspects of what matters to people for the future, on social care, and on future meaningful daily life activities should be included [3].

LVdB and CD developed a first version of the ‘Thinking Now About Later’ tool, involving the expertise of the project management group (FM, CD, TS, LP and LVdB) who made all final decision about content and design of the website [13]. As part of the iterative user-centred and stakeholder-informed design, the tool was reviewed by an advisory group composed of people with dementia, family caregivers, health professionals, and representatives from dementia associations and was tested with several groups of research participants including people with dementia and family caregivers [13].

Divided into nine sections, the ‘Thinking Now About Later’ tool offers a comprehensive exploration of personal values and preferences related to present and future care. Users can navigate all sections through prompts addressing key aspects, such as current and future priorities (e.g. your health, your independence, daily activities that are important to you, what you still want to do in the future, where you reside, social connections, seeing family/ friends/ colleagues regularly, expressing your faith, or experiencing nature or culture), preferences for care and treatments (e.g. consent or refusal of treatments if they would not improve comfort), identification of trusted individuals and/or legal representatives, documentation of preferences through advance directives, and the articulation of any additional considerations important to the person. The tool is meant to be flexible in use, allowing users to skip sections if desired, and acknowledges the subjective nature of responses, highlighting that there are no right or wrong answers. Furthermore, the tool provides practical guidance on next steps, encouraging users to share their preferences with family, friends, and healthcare professionals. For those who are still unsure about what they find important and want for their future care and treatments, the tool suggests using the ‘Life Wishes Cards’ (Levenswensen kaarten in Dutch) as a reflective aid.

An English translation of the ‘Thinking Now About Later’ tool can be found in Appendix 1.

#### Tool 2: the ‘Life wishes cards’ web-based tool

The ‘Life Wishes Cards’ (Levenswensen kaarten in Dutch) are an adaptation of the Go-Wish cards developed in the United States [14]. They serve as a tool to foster conversations on end-of-life preferences for future care through preformulated statements that can be organised according to perceived importance for the user. In our previous work, we undertook the translation and cultural adaptation of the original cards for application in Flanders, Belgium [15]. For inclusion on the ACP support website, we subsequently digitised them.

The ‘Life Wishes Cards’ tool consists of 37 cards, each containing brief statements reflecting preferences for end-of-life scenarios (e.g. “Dying at home”, “Keeping my dignity” or “Being surrounded by my family”). For the digital version, users are asked to categorise the statements into three columns: Very important, somewhat important, or not important. If uncertain, users can place cards on a discard pile. During the process, participants are prompted to reflect on the importance of each statement, envision its role in their future, and consider how their dementia diagnosis may influence their perspectives. If specific priorities are not covered by the preformulated statements, two ‘wild cards’ allow users to add unique considerations that they deem important. The original paper version of the ‘Life Wishes Cards’ also required people to rank the cards they selected as very important to prioritise their 10 top priorities. However, it was found that people with dementia experienced difficulties with such ranking, therefore the digital version of the cards did not include this ranking exercise [15].

The tool serves both reflective and communicative purposes. Users can engage in conversations, explaining their reasoning for each card’s importance, and gather insights from others. Additionally, users can save and print their selections, providing a tangible resource for discussions with others, including healthcare professionals. Users can also come back to their selection and reorganise the cards if they want to.

An English translation of the digitised “Life Wishes Cards” tool can be found in Appendix 2.

### Participants and recruitment

The evaluation study took place in Flanders, the Dutch-speaking part of Belgium, and with Dutch-speaking participants in Brussels, where both Dutch and French are official languages. People with mild to moderate dementia and family caregivers were recruited to the study as dyads; family caregivers were recruited on their own. Eligibility criteria are summarised in Table 1.

**Table 1:** Eligibility criteria

For people with dementia	Being diagnosed with young- or late-onset dementia
**For family caregivers**	Taking active care (physical, emotional, social, etc.) of the person with dementia
**For both groups**	Having an interest in and being willing to try out the ACP website
	Being able to consent to study participation
	Speaking and understanding Dutch
	Having a device that can open the website (computer, tablet, mobile phone)
	Did not participate in the cognitive testing of study materials in a previous study phase
	At least one member of the dyad should be able to navigate the website (e.g. the person with dementia and the family caregiver cannot both have visual impairments or other disabilities preventing them from using the ACP website)

Participants were recruited through regional dementia organisations and neurologists working in two memory clinics. Health professionals were asked to identify and approach potentially eligible participants. If participants expressed an interest in the study, they were referred to the researchers who sent them an information letter and an informed consent form. We organised onboarding sessions where participants were able to discuss their participation in the study, provide informed consent, and were introduced to the ACP support website. Participants were instructed to use the website for eight weeks how and when they preferred. The evaluation study aimed to include a diverse group of participants (i.e. different ages, genders, types of dementia, and relationships between the person with dementia and the family caregiver). A sample size of 30 participants, with 80% of them being dyads was targeted within the evaluation study. Based on previous studies, this sample size was considered sufficient to evaluate feasibility and usability of an interactive website with an heterogenous population [16]. The published research protocol provides a comprehensive account of recruitment strategies [16].

### Data collection

Sociodemographic data, encompassing age, gender, computer literacy, type of diagnosis, and date of diagnosis, was collected through a survey administered at the beginning of the 8-week study period. During the 8-weeks study period, continuous data collection was conducted by logging user activity on the website. This log data was used to record type, frequency, and timeframe of usage of all components and features of the ACP support website. In this study, we focused on the interactions with the web-based reflection and communication tools.

We conducted semi-structured interviews with dyads composed of people with dementia and family caregivers, or with family caregivers alone, to explore their experiences of using the ACP website. Interviews were conducted at the end of the 8 week-study period. The interviews were conducted in Dutch, between October 2022 and May 2023, took place in the participants’ homes and were audio-recorded. The interview questions included questions about participants’ experiences with the different components of the ACP support website including the two interactive ACP tools (Appendix 3). Follow-up questions were asked as needed to clarify participants’ answers. All interviews were completed by the third author (CD) or a research assistant.

### Data analysis

Descriptive statistics were used to analyse participants’ sociodemographic characteristics, using SPSS. To analyse the log data of the interactions with the web-based tools, we used R. The data was summarised using descriptive analysis.

All interviews were recorded and transcribed verbatim. All transcripts were pseudonymised. We conducted thematic framework analysis [17], with the assistance of the qualitative analysis program NVivo. The process of thematic framework analysis involves several key stages. They encompass data familiarisation, the development of a thematic framework, indexing all data against this framework, charting to condense the data, and finally, mapping an interpretation [17]. Transcripts were read through while listening to the audio recording. FM and CD then read and re-read the transcripts to familiarise themselves with the interview data. Next, analysing a subset of 20% of the interviews, they established subcodes based on the interview guide and created new subcodes when necessary, forming a preliminary framework for analysis. The two researchers then compared their coding, and differences were discussed and resolved as to agree to a definite framework. FM and CD then systematically applied this framework to all the interview transcripts. In the final step, data were abstracted to create final themes. The researchers reviewed the final themes to reach consensus in the interpretation of the data.

### Ethics

This study received approval from the Ethical Review Board of Brussels University Hospital of the Vrije Universiteit Brussel on 07 October 2022 (B.UN 1,432,022,000,179), and all participants provided written informed consent. We obtained informed consent from potential participants using a double-consent approach (i.e. consent for patients’ participation is signed both by themselves and by their family caregivers acting as witnesses). The researchers ensured participants’ understanding of the study and their rights by engaging in discussions regarding the information presented in the informed consent form with both people with dementia and their family caregivers [16].

## Results

In total, we included 52 participants in the study, of which 21 were people with dementia and 31 were family caregivers of people with dementia. All people with dementia participated together with their family caregivers, and 10 family caregivers participated on their own. Reasons for participating on their own were that the person with dementia: (1) was unable to provide consent to participate in the study, (2) did not wish to participate in the study, (3) recently moved to a nursing home, or (4) did not want to discuss ACP. An overview of the participants’ characteristics is provided in Table 2.

**Table 2:** Sociodemographic characteristics of people with dementia and family caregivers

Number of people with dementia		21
Age, mean (SD)		62.1 (10.9)
Age range		50–78
Gender, n (%)		9 female (43), 12 male (57)
Type of diagnosis, n (%)	Alzheimer’s disease	15 (71)
	Vascular dementia	1 (5)
	Frontotemporal dementia	3 (14)
	Lewy body dementia	1 (5)
	Unknown	1 (5)
Highest education level, n (%)	Primary school	6 (29)
	High school	5 (24)
	Applied sciences	7 (33)
	University	3 (14)
Profession, n (%)	Employed	1 (5)
	Retired	20 (95)
Relationship to caregiver, n (%)	Partner	18 (86)
	Parent (in law)	3 (14)
Self-rated computer skills, mean (SD), scale range: 0–10		4.2(3.1)
**Number of family caregivers**		**31**
Age, mean (SD)		62.8 (10.4)
Age range		34–84
Gender, n (%)		21 female (68), 10 male (32)
Type of diagnosis of relative, n (%)	Alzheimer’s disease	20 (64)
	Vascular dementia	3 (10)
	Frontotemporal dementia	3 (10)
	Lewy body dementia	1 (3)
	Parkinson’s dementia	1 (3)
	Unknown	3 (10)
Highest education level, n (%)	Primary school	3 (10)
	High school	5 (16)
	Applied sciences	14 (45)
	University	9 (29)
Profession, n (%)	Employed	16 (52)
	Retired	15 (48)
Relationship to person with dementia	Partner	25 (81)
	Son/daughter (in law)	6 (19)
Self-rated computer skills, mean (SD), scale range: 0–10		7.5 (2.1)

### Frequency and type of use of the web-based reflection and communication tools

During the 8-week period, the ‘Thinking Now About Later’ tool and the ‘Life Wishes Cards’ tool were visited a total of 136 and 91 times, respectively. The time spent per session on one of the tools ranged from 1 to 90 min, for an average of 14 min (SD = 27.45). The log data showed that, of the 52 participants, 22 actively used the web-based tools, i.e. sorting cards in the ‘Life Wishes Cards’ tool and filling in the ‘Thinking Now About Later’ tool. Among participants who used the web-based tools, 15 only used them once. Seven participants revisited one of the tools at another time.

### Perceived usefulness of the web-based reflection and communication tools

In the interviews, those who had used the tools mentioned that they particularly valued the web-based tools, i.e. the “Life Wishes Cards” tool and the “Thinking Now About Later” tool. Both people with dementia and family caregivers highlighted the inherent value of such tools in providing guidance for supporting ACP conversations between people with dementia and their family caregivers. This guidance was seen as a way to eliminate the need for users to independently generate topics for discussion. They appreciated that the web-based tools provided concrete examples and scenarios to discuss and welcomed the interactive aspects of the tools such as the opportunity to fill in boxes or sort statements according to importance.

That’s precisely the added value of that website, you know. That you have tools - that you don’t have to come up with things yourself about what we’re going to talk about this time. You have a tool. You have a structure. That is important because otherwise you are a bit unfocused - or not really unfocused, but… Now it’s really… There’s a guiding line to it. That’s good. – person with dementia #21.

Furthermore, family caregivers noted that the tools were valuable for their family member living with dementia, as they allowed them to express thoughts that they deemed significant but struggled to communicate. The tools allowed participants to identify topics that they found important to discuss and gave them the opportunity to start these discussions. One family caregiver mentioned that while using the cards, her partner had emphasised the importance of discussing death and dying and admitted that he had rarely engaged in such discussions. The card tool on the website provided the prompt needed to open the conversation about this topic.

“He also mentioned that he has always considered discussing death important. He feels that he doesn’t do it enough. And there was actually a card in there [in the Life Wishes cards] about ‘Talking about what death means to me,’ that was something on that card.” – Family caregiver #8.

Finally, both people with dementia and family caregivers mentioned that they found it important to be able to revisit the web-based tools and not only use it once. They noted that it would not be sufficient to only use it once, as ACP topics required a significant amount of reflection and communication. Fourteen participants saved the results of their first-time use with the intention of revisiting their preferences and perhaps adapting them based on new information provided on the website or by health professionals. They also noted that they would like to keep using the tools after the study period.

We filled it [the Life Wishes cards] out at the beginning and then filled it out again after the 8 weeks. By actually reading everything and understanding how to approach these topics, there were some aspects that I used to think were unimportant, that I now find important. – Family caregiver #26.

### Perceived barriers to using the interactive reflection and communication tools

Although all participants who used them found both the web-based tools useful and user-friendly, some family caregivers noted that they could be more concrete. They found the tools a good way to initiate conversations but were unsure about what the next step should be once they had discussed the different prompts on the ‘Life Wishes Cards’ tool or the ‘Thinking Now About Later’ tool. Furthermore, five participants, both people with dementia and family caregivers, also pointed out that there were many prompts and it felt like they would never be done, which could be frustrating. They compared it with the fact of filling in an advance directive, which they argued can be more satisfying as it could lead to having the feeling of a completed document and the feeling of having their affairs in order. They mentioned that with the use of the web-based tools, they remained in a state of reflection, which could give them the feeling of an uncomplete process and ‘not being finished’.

“Well, I have the sense of ‘That’s not finished yet.’ But that probably won’t be possible, right? But at some point, you want such a finished document, where you say, ‘We’ve discussed that enough now; it’s done!’ And then you save it somewhere or print it out once, for example, to discuss it with the children. But I don’t have the feeling now of ‘We’ve gone through it completely.’ Maybe I haven’t gone into it deeply enough… I don’t know how to explain it, but I don’t think you can do it in one go. I want to look at it a few more times, so to speak, to see, ‘Is that what you want? Are you sure?” – Family caregiver #2.

Although no technical difficulties were reported, people with dementia preferred to use the website together with family caregivers due to a lack of confidence and out of fear of doing something wrong. Several family caregivers also reported that their family member with dementia would not be able to use the website on their own. This was either because the stage of dementia was too advanced (i.e. people with moderate dementia), or because of a lack of digital skills. In the case that family caregivers felt the person with dementia struggled to use the web-based tools, family caregivers took the lead in the use of the website and guided their loved ones through the web-based tools. Some family caregivers explained the measures they took to use the interactive tools together with their family members with dementia and support them in using these tools. For both tools, but especially the ‘Life Wishes Cards’ tool, they read each prompt out loud and clearly asked them whether they found it important, somewhat important, or not important. While these dispositions seemed to facilitate and enhance the participation of people with dementia in some instances, in other cases family caregivers noted that it did not result in in-depth conversations, or that they needed to ask several small follow-up questions to help clarify the preferences of their family members with dementia.

“I start with - ‘What’s your reaction when you see that card?’ And then organising, how important do you find it? If it’s very important, then I try to confirm with a few more questions what he meant.” – Family caregiver #16.

Finally, five family caregivers and people with dementia used the interactive tools together and then mentioned that they would wait until their appointment with their doctor before revisiting them. They mentioned that they may gain new insights into possible treatments and that would help them to revisit their preferences using the web-based tools. Thus, discussions with health professionals seemed to be seen as an important facilitator or support in the use of the web-based tools.

“After using the interactive tools, we now have a list of what he finds important - occasionally, I do say, ‘We’ll take another look at that.’ We’ve created a sort of ranking from 1 to 10 of the things he finds the most important. We need to go to the doctors in February, where we can discuss possible treatments that he would and wouldn’t undergo. Afterwards we’ll try again to see, ‘Is it really the order you deemed important?” – Family caregiver #19.

## Discussion

In this study, we described and evaluated two web-based reflection and communication tools on ACP for people with dementia and their family caregivers, namely the ‘Thinking Now About Later’ tool and the ‘Life Wishes Cards’ tool. Both are grounded in a ‘what matters most to you now and in the future’ approach and aim to provide a flexible way to support ACP within the family context. Our results showed that more than half of the participants that used the ACP support website did not use the web-based reflection and communication tools. However, for those that did use the tools, they were perceived as useful and provided a framework or guidance for people with dementia and family caregivers to engage in ACP conversations. The ‘Thinking Now About Later’ tool and the ‘Life Wishes Cards’ tool (Levenswensen kaarten in Dutch) encouraged people with dementia and family caregivers to think about and talk about their preferences for current and future care and medical treatments from the perspective of ‘what matters most’ to them. This ‘what matters most’ approach was well received by participants in our study, and they found it a useful way to engage in ACP conversations. Barriers to use included a lack of concrete steps to take once the web-based tools were completed. Furthermore, participants experienced challenges with the use of the ‘Life Wishes Cards’ particularly, as people with dementia sometimes had difficulties using them on their own and family caregivers had to assume a facilitating role by explaining the pre-formulated statements to encourage reflection and communication.

Out of the 52 participants, 22 actively used the web-based reflection and communication tools. The ACP support website on which the web-based reflection and communication tools were embedded had two main goals: providing information about ACP and supporting initiation and engagement in ACP conversations between people with dementia and family caregivers. The web-based tools were mainly developed to address the second aim. In both the development study and the evaluation study of the ACP support website as a whole, we found that people with dementia and family caregivers navigated the website in a flexible manner (i.e. some only read the information provided on the website, only used the advance directives provided on the website, or preferred to use the web-based tools more intensively) [13, 18]. This flexible navigation is meant to accommodate diverse ACP readiness levels observed in people with dementia and family caregivers [10, 19–21] and thus may explain why half of the participants actively used the tools and the other half did not. People with dementia experienced barriers to using the web-based tools, with family caregivers stating that this was due to either a lack of digital skills or due to the cognitive decline associated with dementia. Family caregivers often took the lead in the use of the tools, adopting different strategies to include the person with dementia through simplified explanations of the content of the web-based tools or prompts and follow-up questions to stimulate participation from the person with dementia. This is consistent with previous studies that emphasise the significant contribution of family caregivers as primary providers of support and guidance for people with dementia [22, 23]. In the context of the use of technology particularly, family caregivers frequently play a vital role in ensuring accessibility and overcoming challenges to technology use [22, 23]. However, in instances where people with dementia may heavily rely on the digital skills of family caregivers, it could potentially place an added burden on the family caregivers [24]. This also implies a lower accessibility to the tools for people with dementia who do not have family caregivers or other people who can help them with this. There is a need to explore strategies to address potential support needs of people with dementia, while also ensuring that family caregivers are adequately supported in their facilitating roles.

However, the challenges experienced by people with dementia, especially with the ‘Life Wishes Cards’, may not be solely due to the web-based nature of the tool. Paper-based card sorting tools have been shown to be effective in eliciting preferences for people with dementia [14, 15, 25, 26], yet they can present similar challenges to those encountered in our study. Previous research into eliciting ACP preferences with card tools has found that a more thorough facilitation process may be required depending on factors such as cognitive decline, impaired sight, or loss of motor skills [27]. This may include turning statements into questions for the person with dementia or revisiting the use of the cards at a later time.

A lack of concrete steps to take after the completion of the web-based tools was pointed out by some participants. Although the web-based tools explicitly encouraged users to communicate preferences with family members, friends, and health professionals, some of our participants mentioned lacking guidance on the next steps to take after having used the web-based tools and lacked the feeling of having finished or completed the ACP process. This finding might be related to the idea among some participants that there always needs to be a concrete product such as an advance directive in an ACP process. It might also be related to the need of some people to have tangible and concrete outcomes or outputs when engaging in a planning process. Earlier research has found that people with dementia and family caregivers often associate ACP with medical planning, often through advance directives [28]. However, it should be noted that some participants in our study did describe the concrete steps they would take after having used the tools i.e. bring the results from their use of the tools to their next medical appointments to discuss them with their healthcare providers. This again shows the difference between people in how they approach an ACP process and what they find supports this process. It also highlights the potential role of health professionals in supporting the use of the web-based reflection and communication tools and providing more concreteness after the use of the web-based tools.

The web-based reflection and communication tools included in our study are the first to address the specific ACP needs of people with dementia and family caregivers. The evidence-based nature of the tools, rooted in international ACP literature and cultural adaptation processes, positions them as valuable resources. Whilst the local legal and regulatory context influenced some of the content of the two web-based reflection and communication tools, the ‘what matters most’ approach and the flexible navigation adopted in the web-based tools can be widely applicable. A recent systematic review has shown that most web-based ACP tools available to the public do not provide information about their development process, are not evidence-based, and are not evaluated in a study [29]. Our study provided a transparent and detailed description and evaluation of the two web-based ACP tools.

A few limitations need to be considered in this study. Firstly, the interviews involved family caregivers on their own or joint interviews with both the person with dementia and their family caregivers. There may be a risk of the family caregiver perspectives overshadowing those of the people with dementia, possibly leading to an incomplete understanding of the latter’s experiences. Additionally, while efforts were made to include a diverse sample, participants predominantly represented a highly educated demographic.

Future research should further evaluate how people with dementia and family caregivers use the web-based communication and reflection tools and their potential role in discussing ACP with health professionals. Additionally, researchers should focus on how to best support the use of the web-based reflection and communication tools, and whether an element of human interaction, such as a training, could effectively support people with dementia and family caregivers.

## Conclusion

This study presented and evaluated two web-based reflection and communication tools to support ACP for people with dementia and family caregivers, the ‘Thinking Now About Later’ tool and the ‘Life Wishes Cards’ tool, which were part of an ACP support website. While less than half of the participants visiting the ACP support website used the web-based reflection and communication tools, those who did use them had positive perceptions regarding their usefulness. We identified certain barriers in the use of the web-based tools, including a lack of concrete steps to take after completion of the tools and challenges in practical usage for some people. The study also highlighted the pivotal role of family caregivers as facilitators in using the web-based tools and the need for tools that allow flexible use tailored to people’s needs.

### Electronic supplementary material

Below is the link to the electronic supplementary material.

Supplementary Material 1

## Data Availability

The datasets generated and/or analyzed during the current study are not publicly available due to restrictions applied to the availability of this data but are available from the corresponding author on reasonable request.

## Abbreviations

ACP: Advance care planning
