# Supplementary material for: Evaluation of interactive web-based tools to stimulate reflection and communication about advance care planning with people with dementia and their family caregivers
Source: BMC Palliat Care. 2024 Jun 28;23:162. doi: 10.1186/s12904-024-01486-4 (PMC11212172; doi:10.1186/s12904-024-01486-4)
Supplement: Supplementary file 1 — Supplementary Material 1 [file 12904_2024_1486_MOESM1_ESM.docx]

**Appendix 1: English translation of the *“Thinking Now About Later” tool***

**
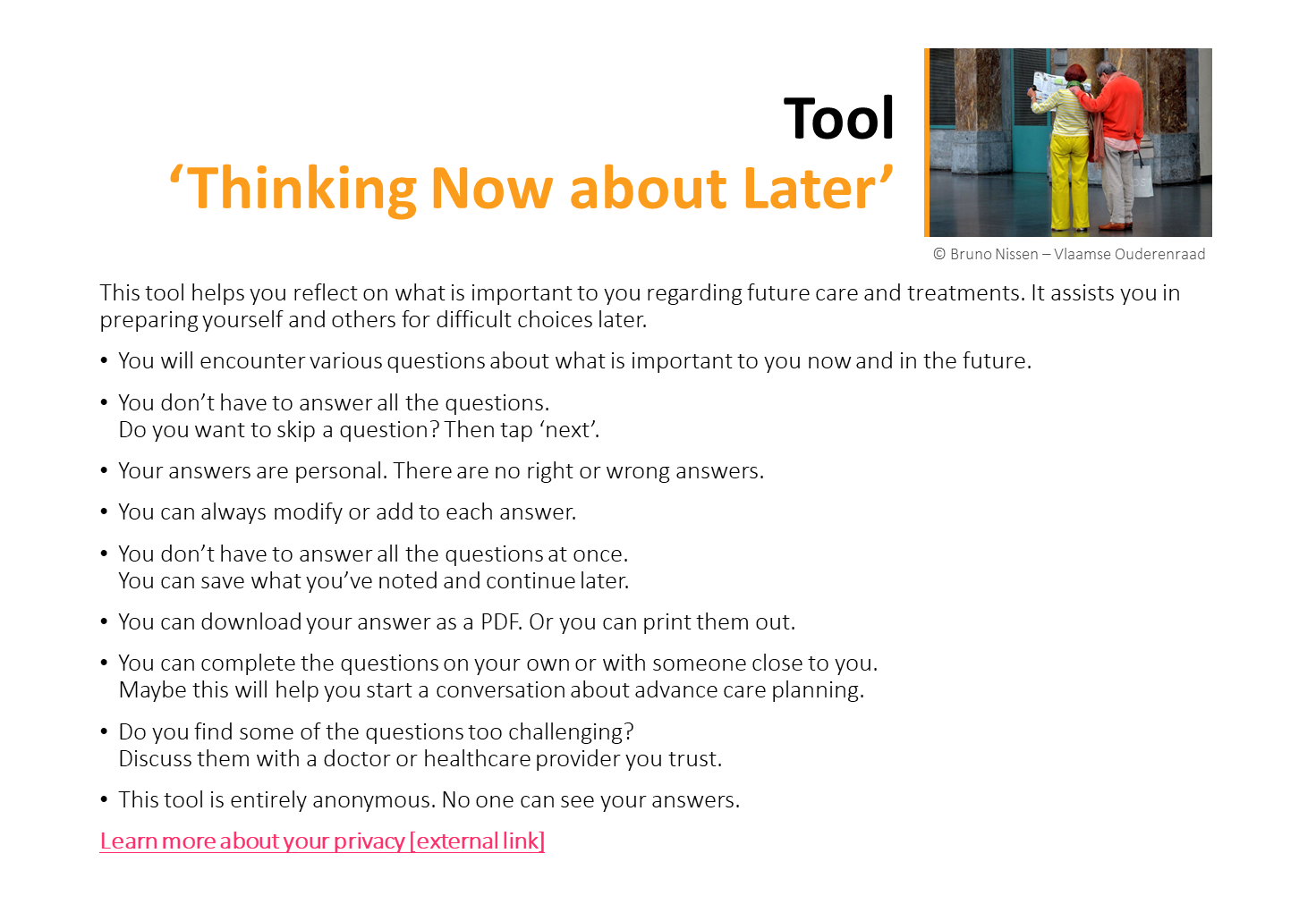
**

**
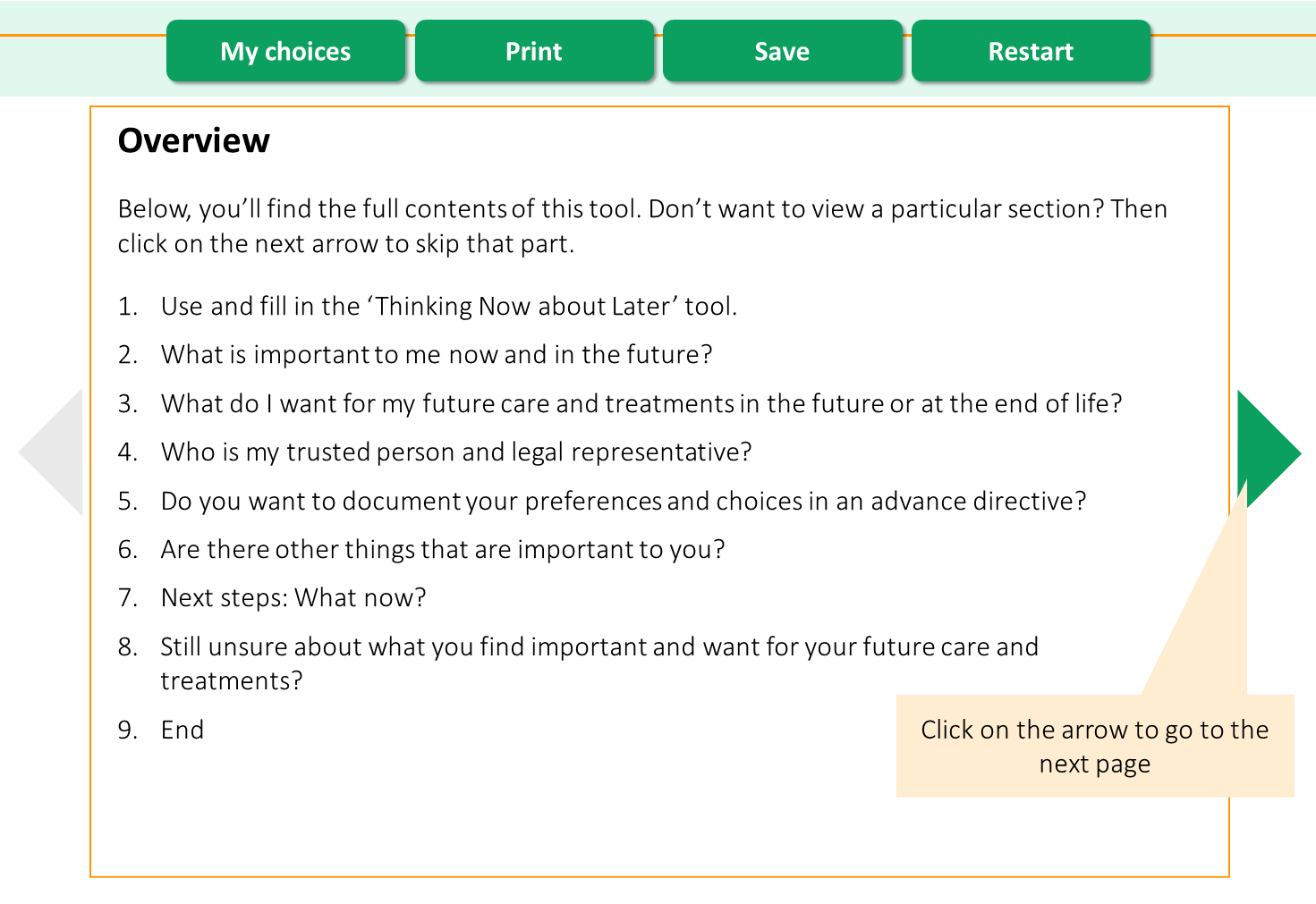
**

**
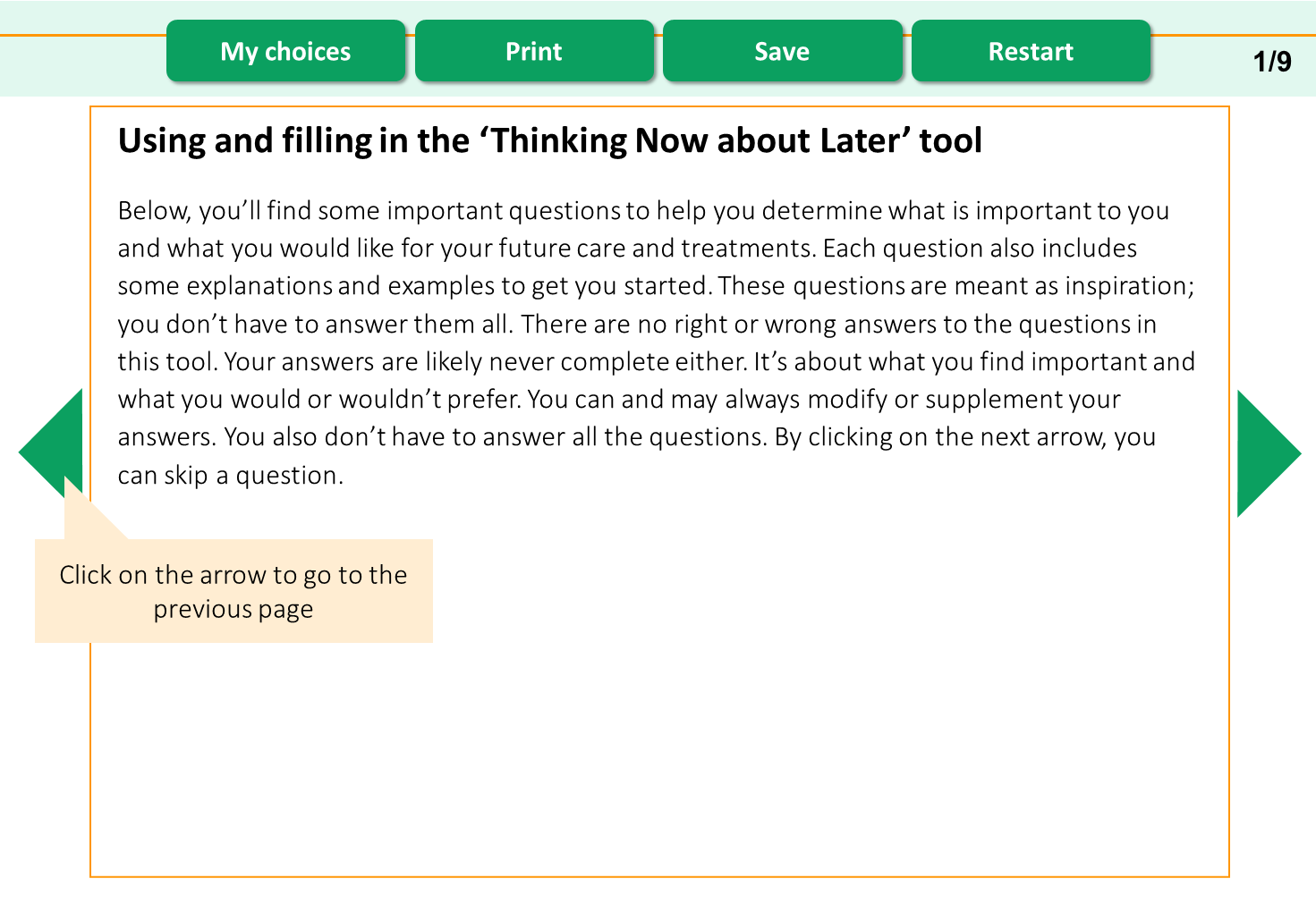
**

**
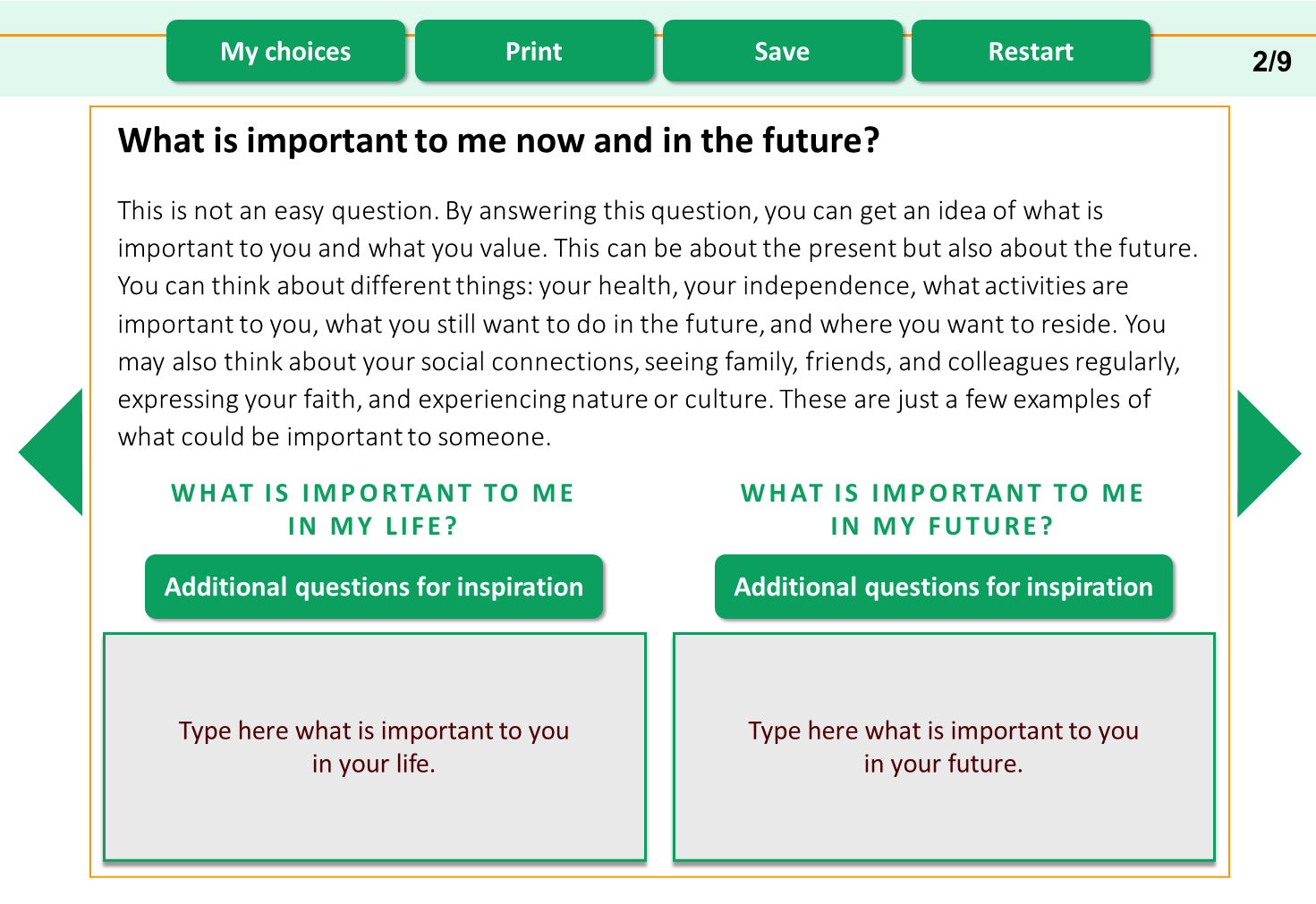
**

**
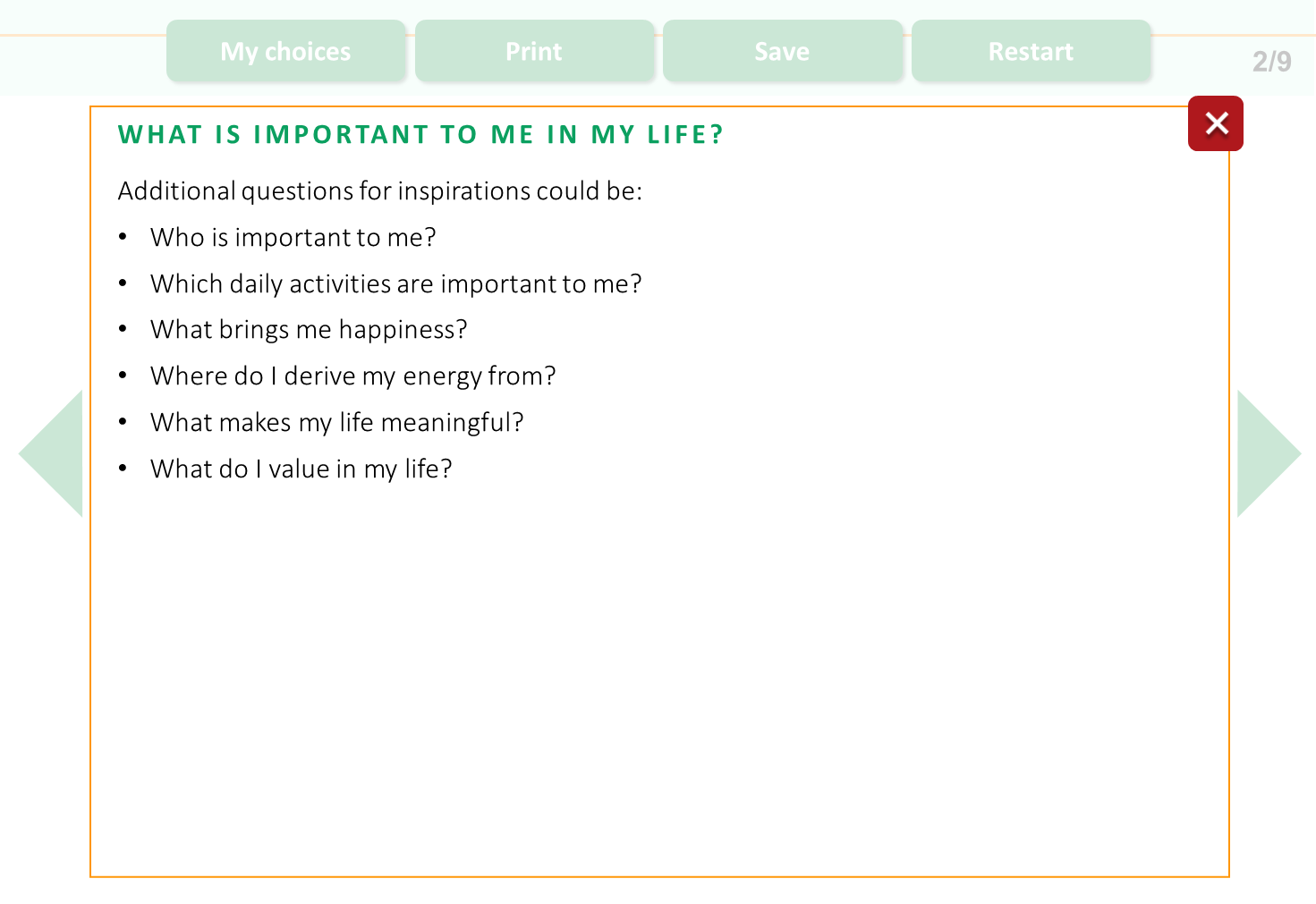
**

**
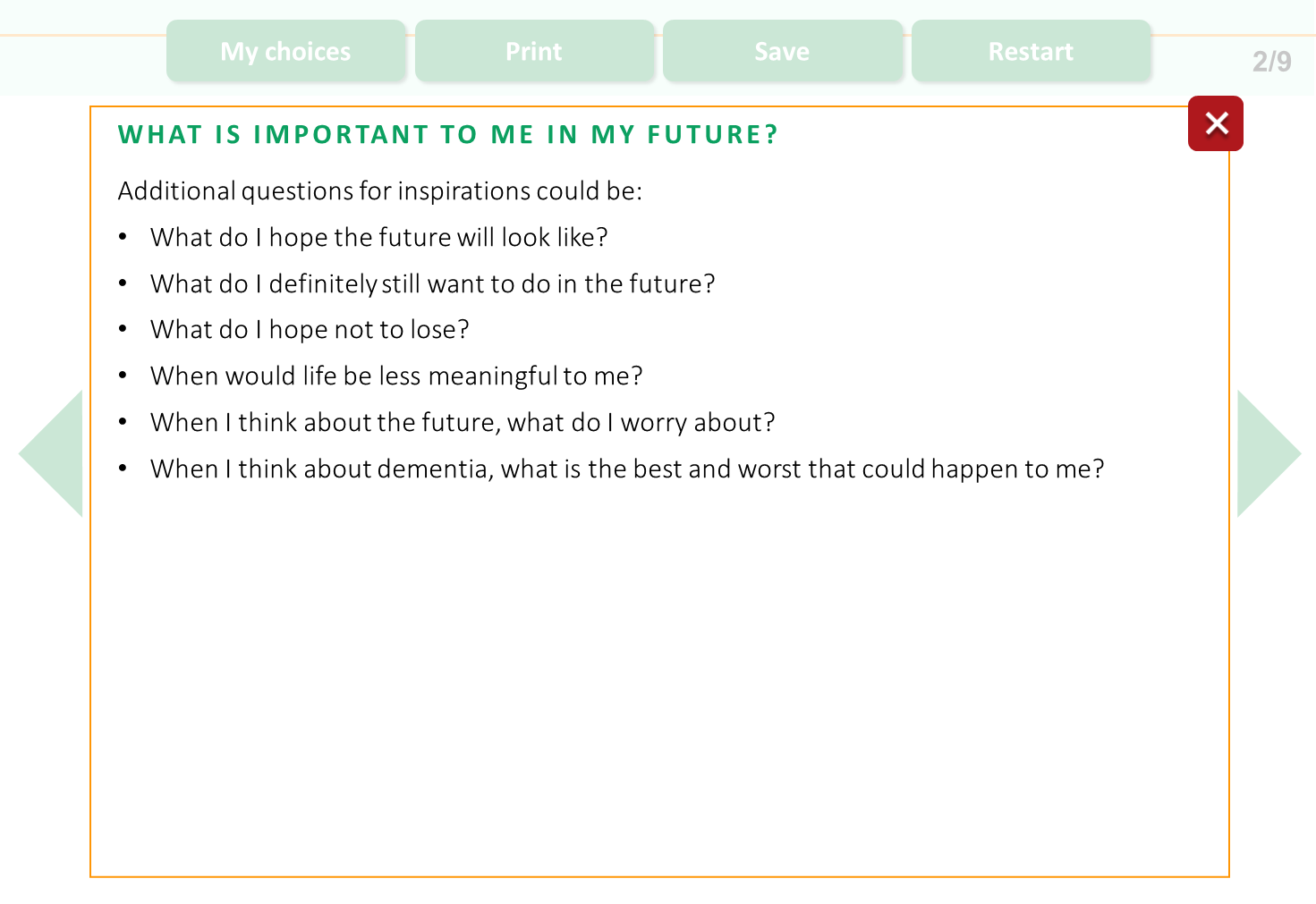
**

**
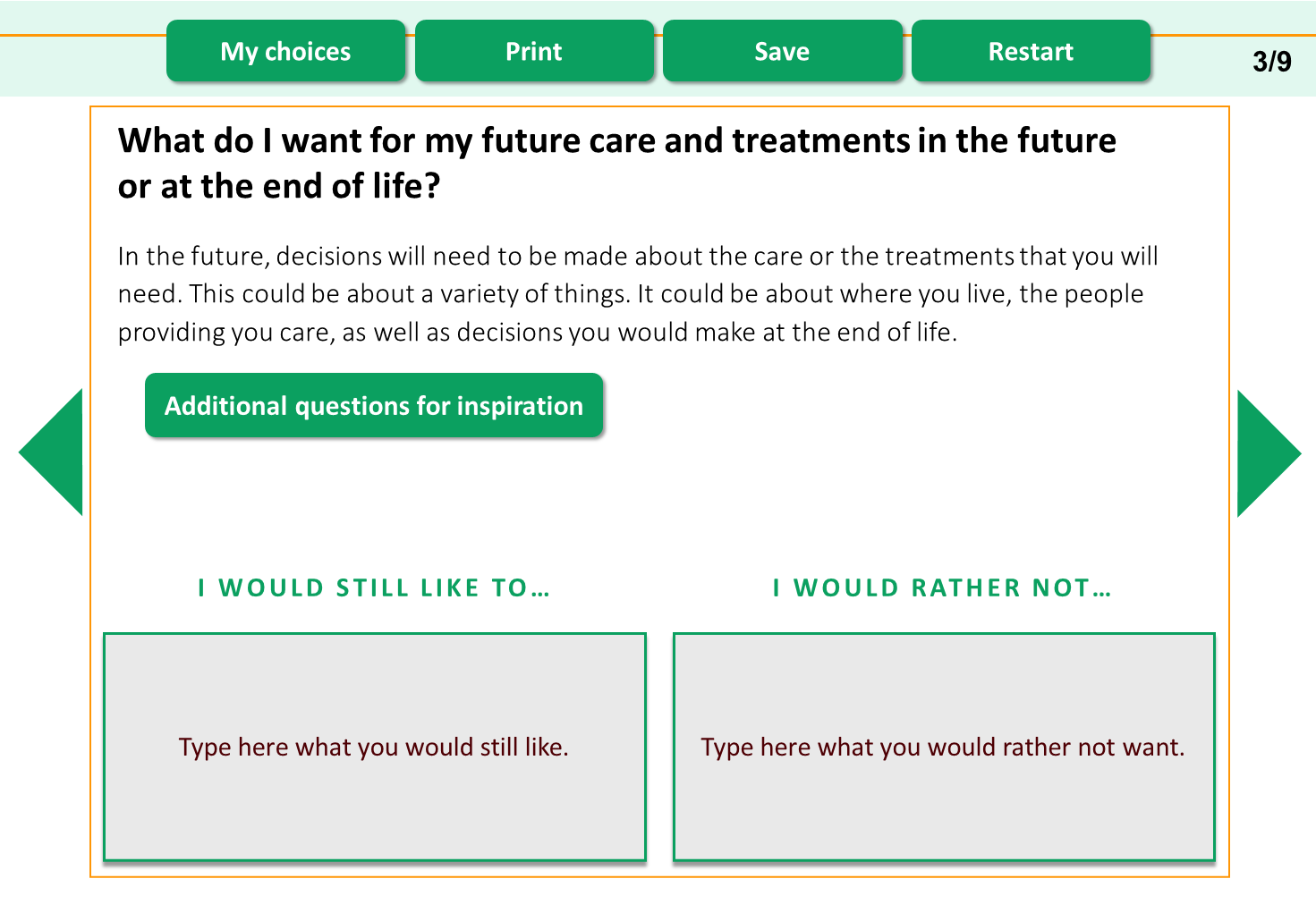
**

**
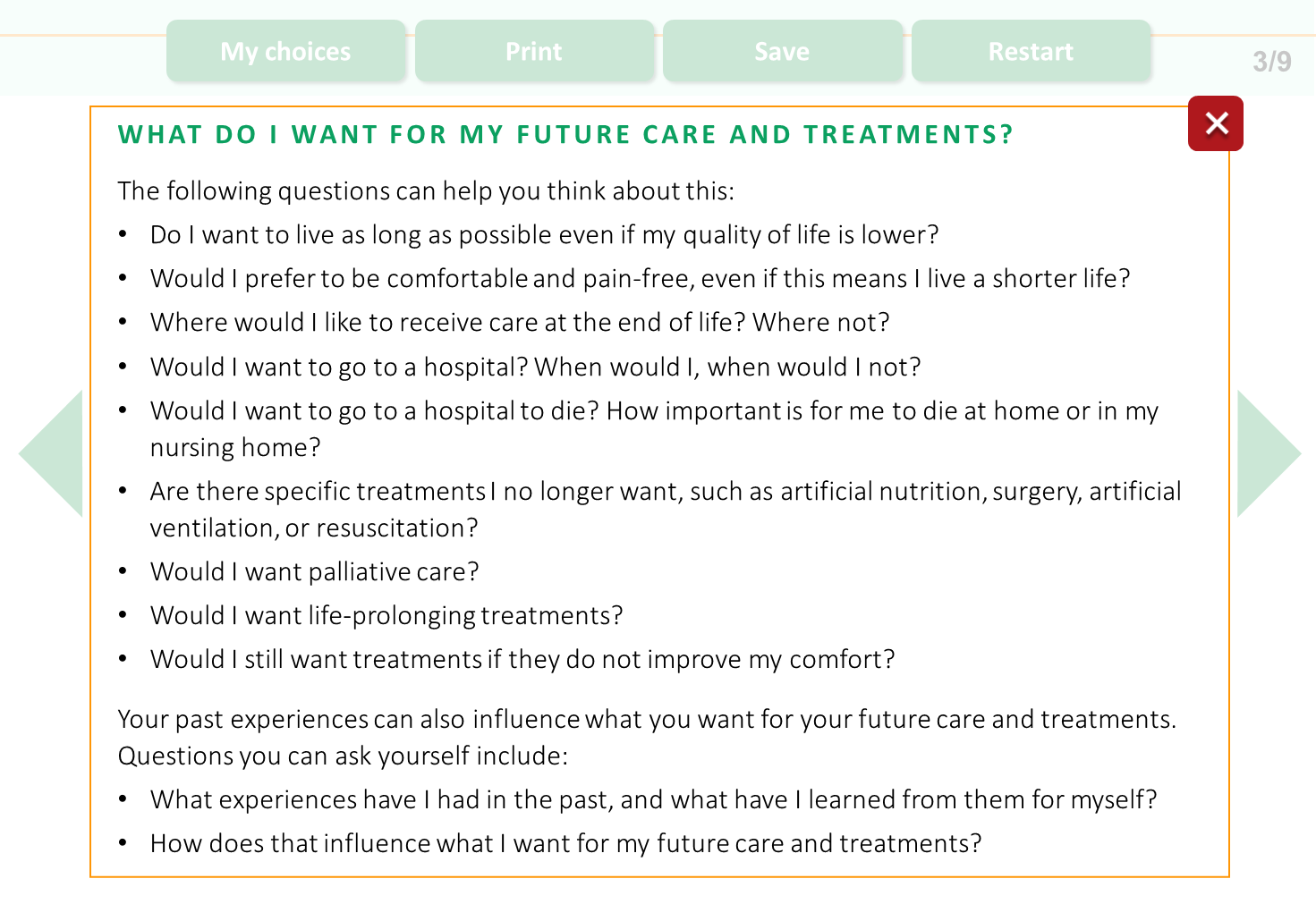
**

**
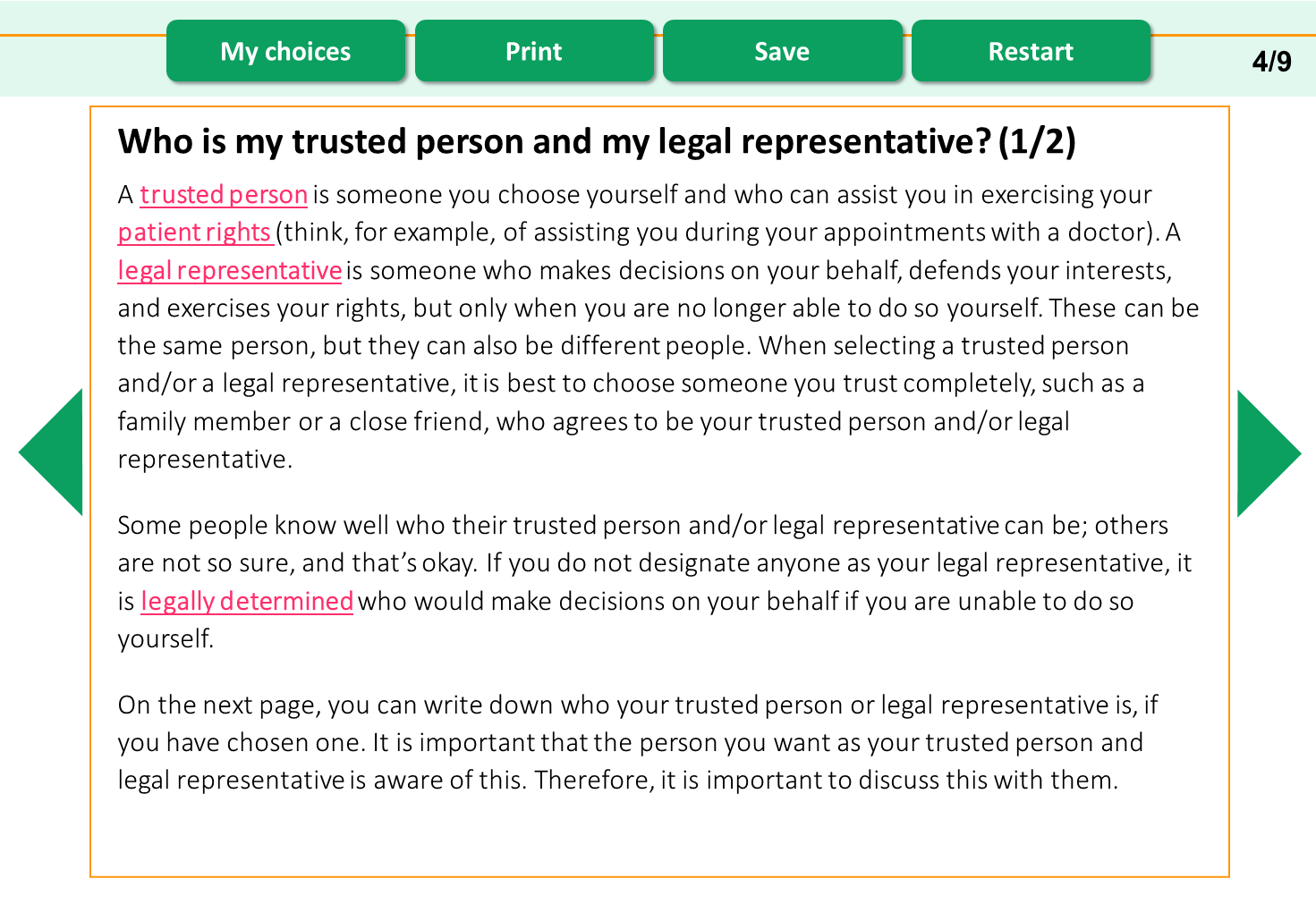
**

**
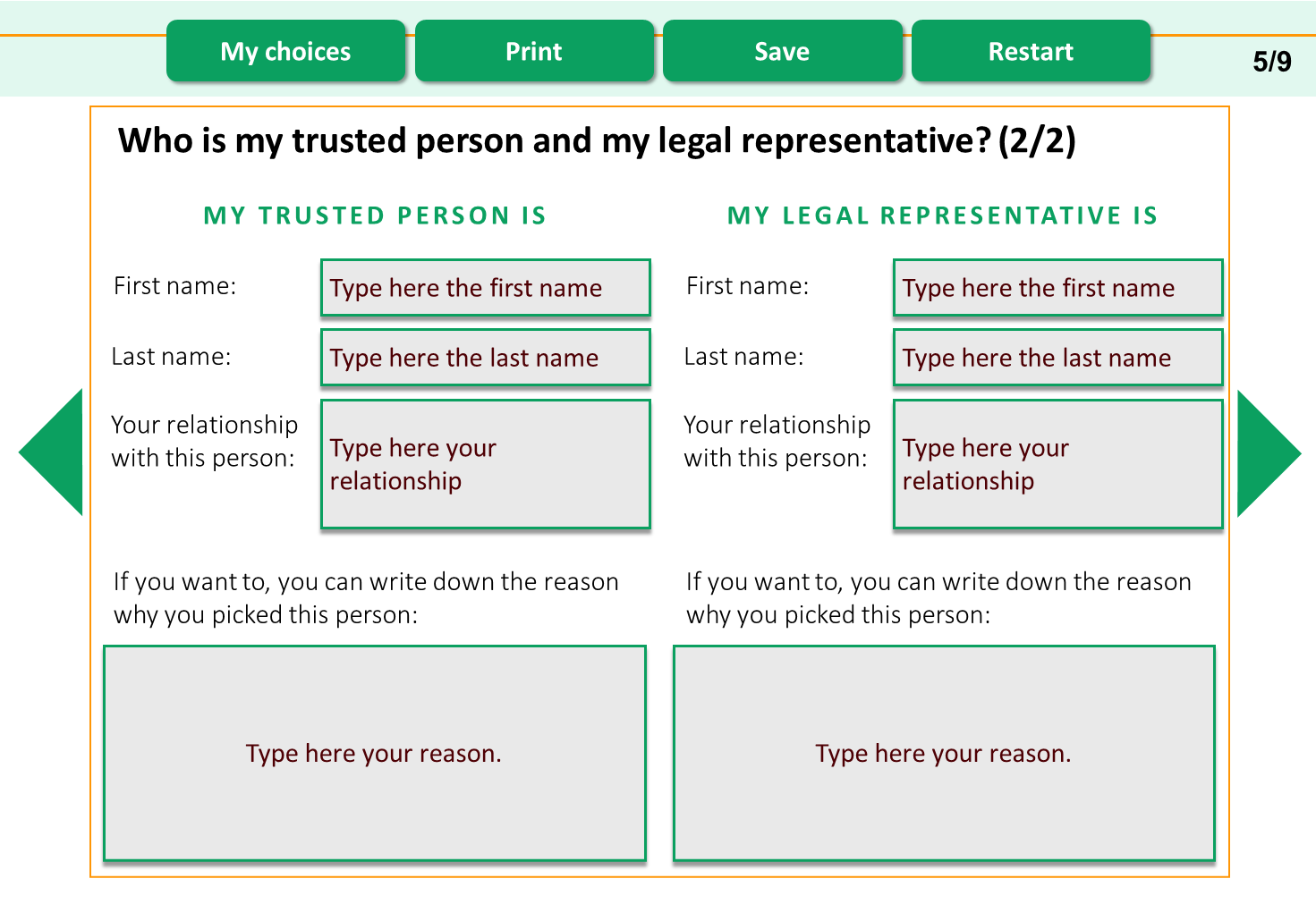
**

**
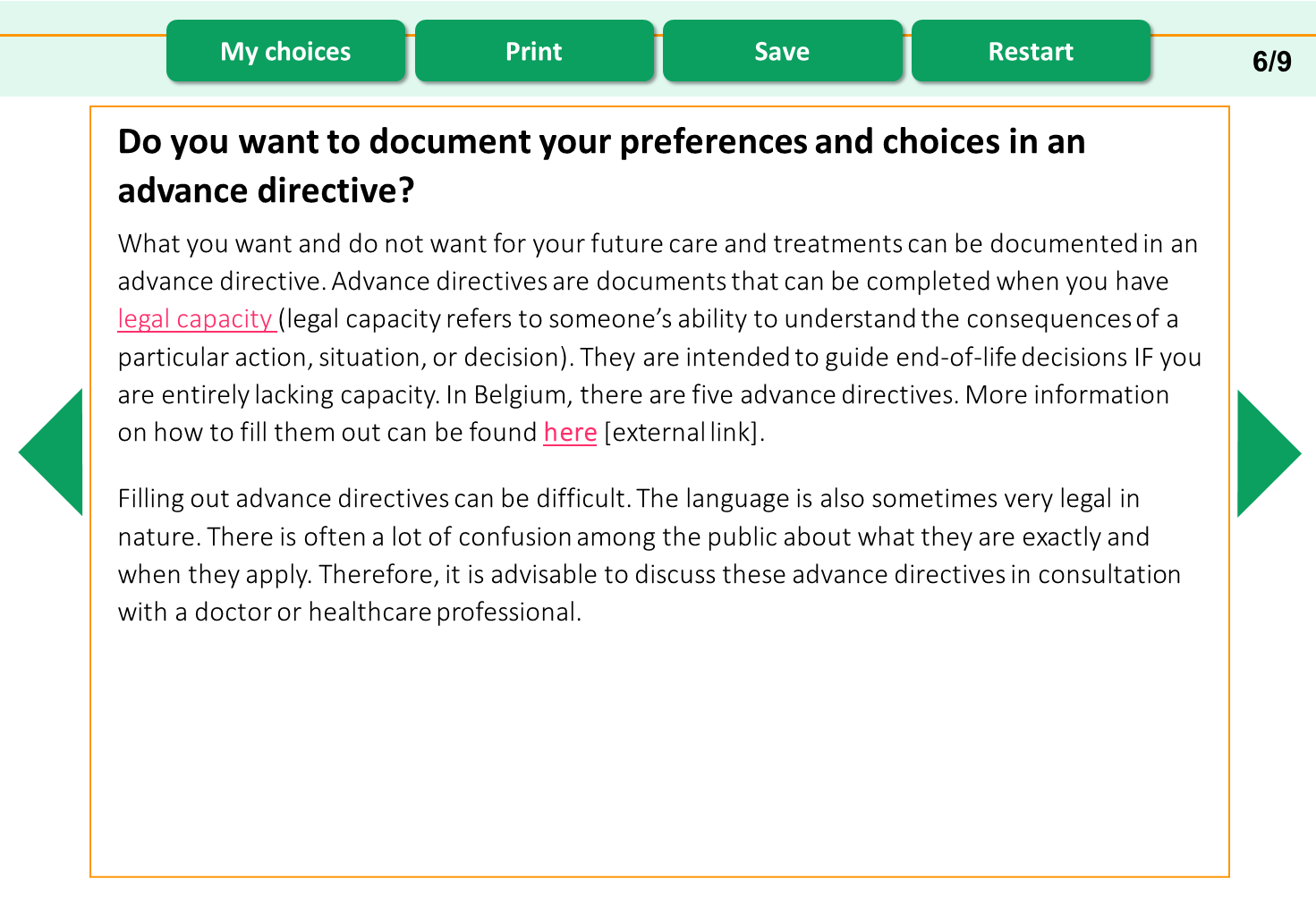
**

**
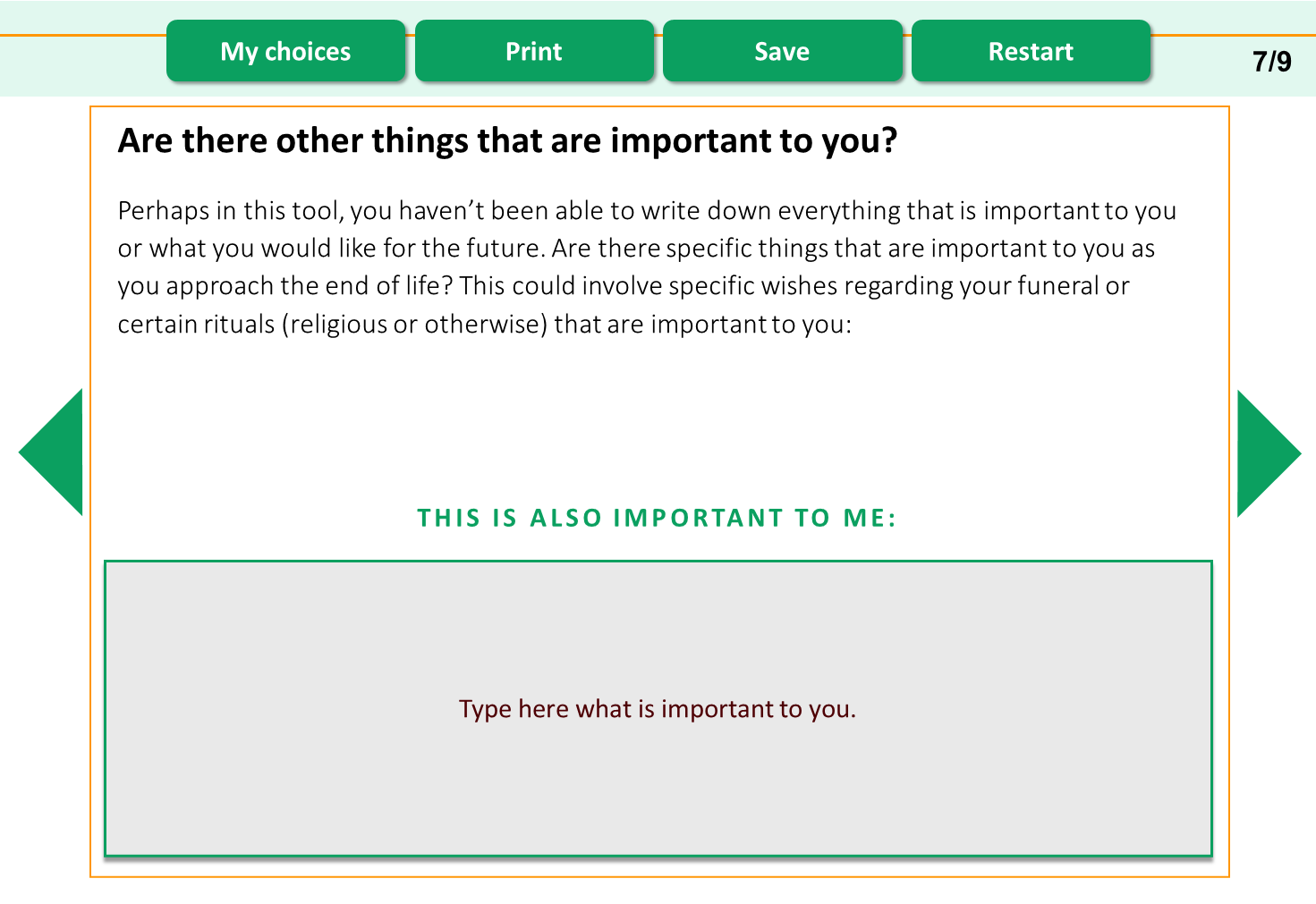
**

**
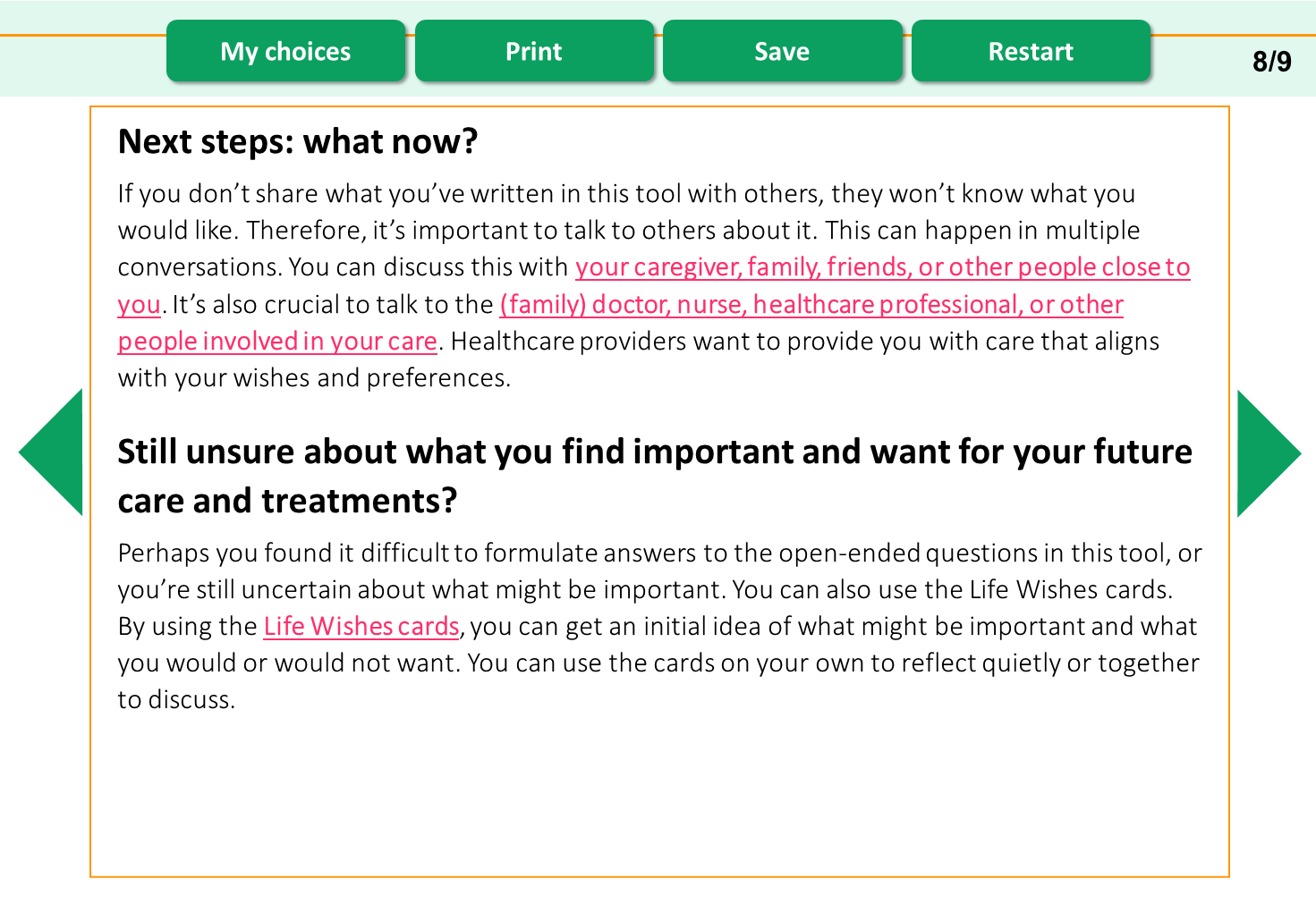
**

**
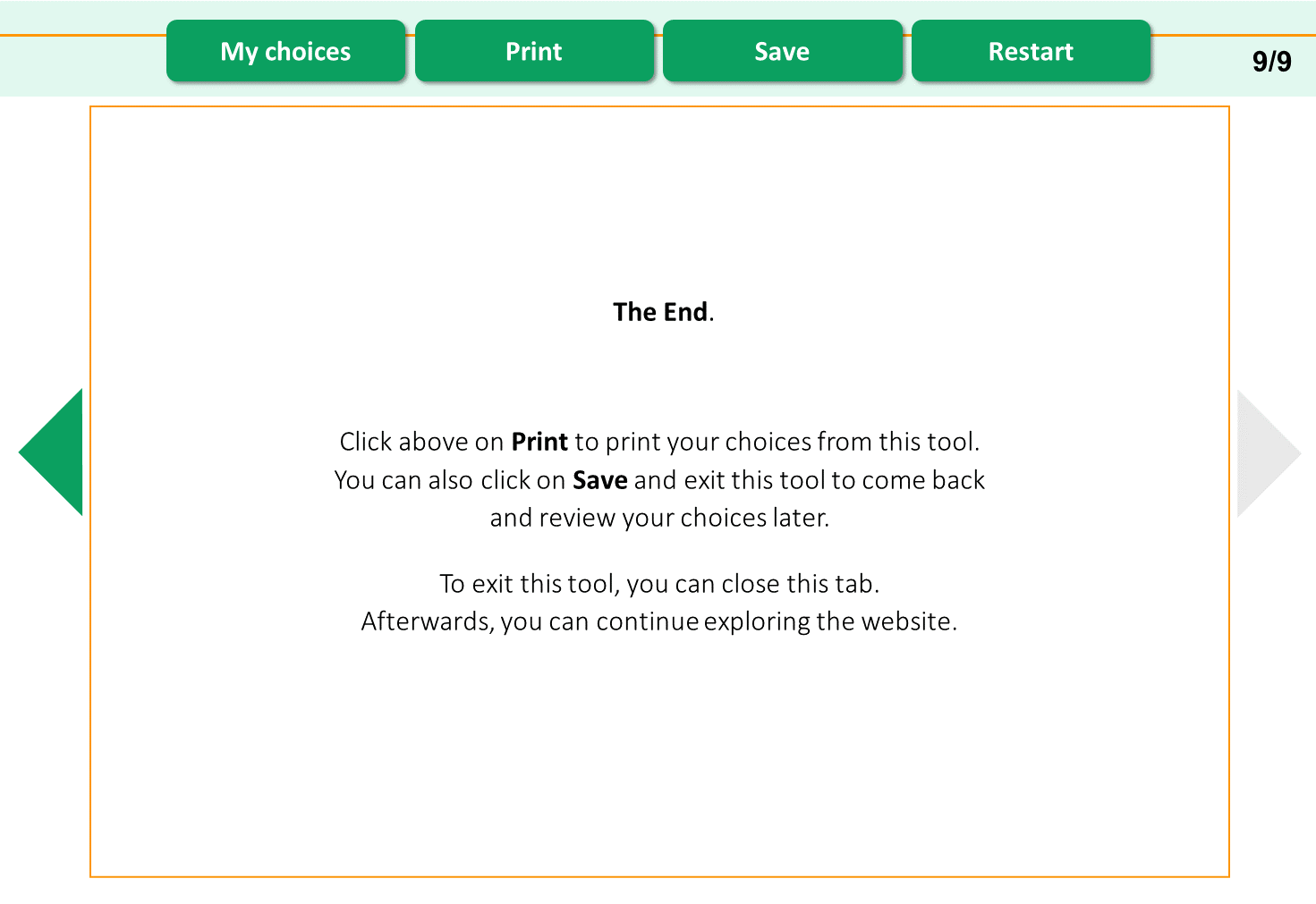
**

**Appendix 2: English translation of the “Life Wishes Cards” tool**

**
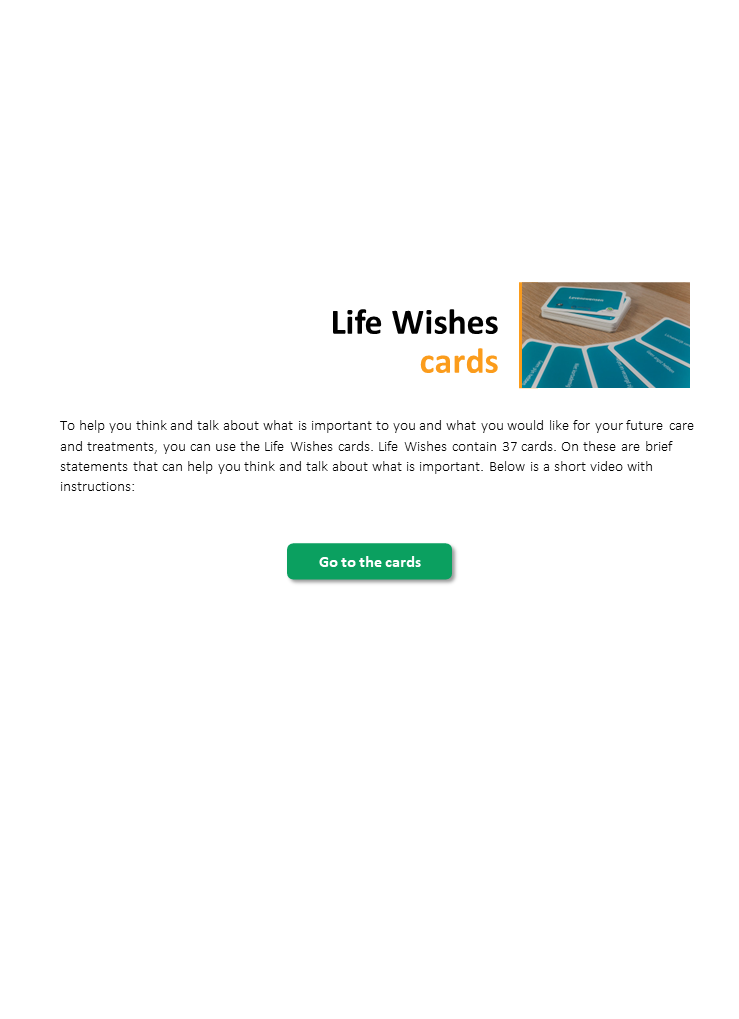
**

**
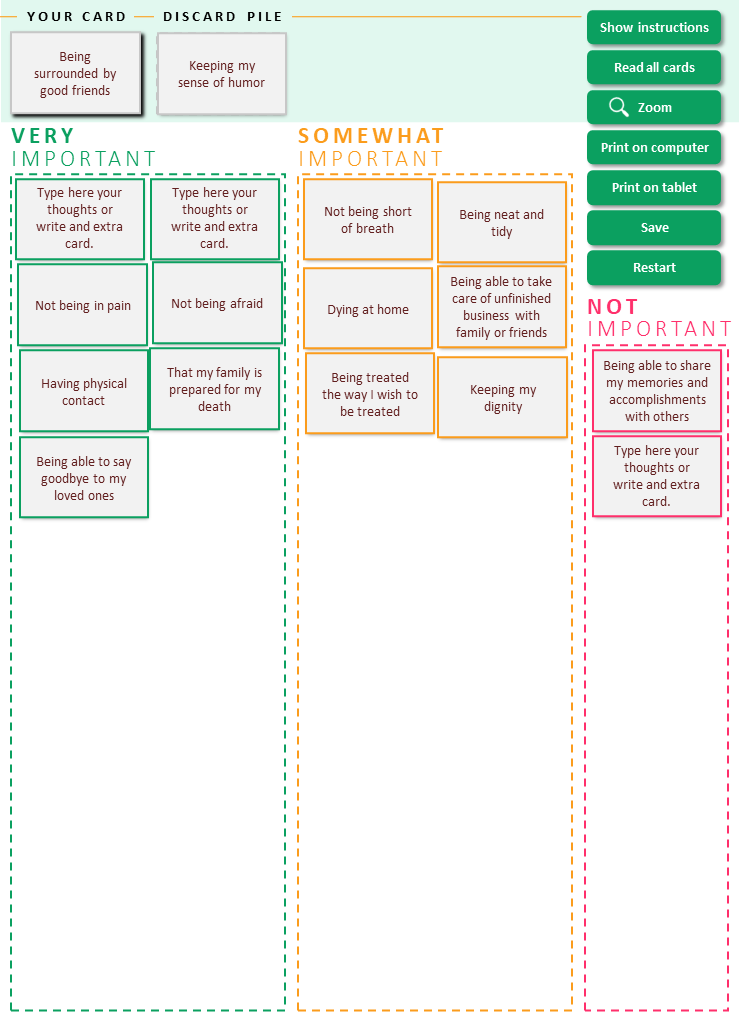
**

**
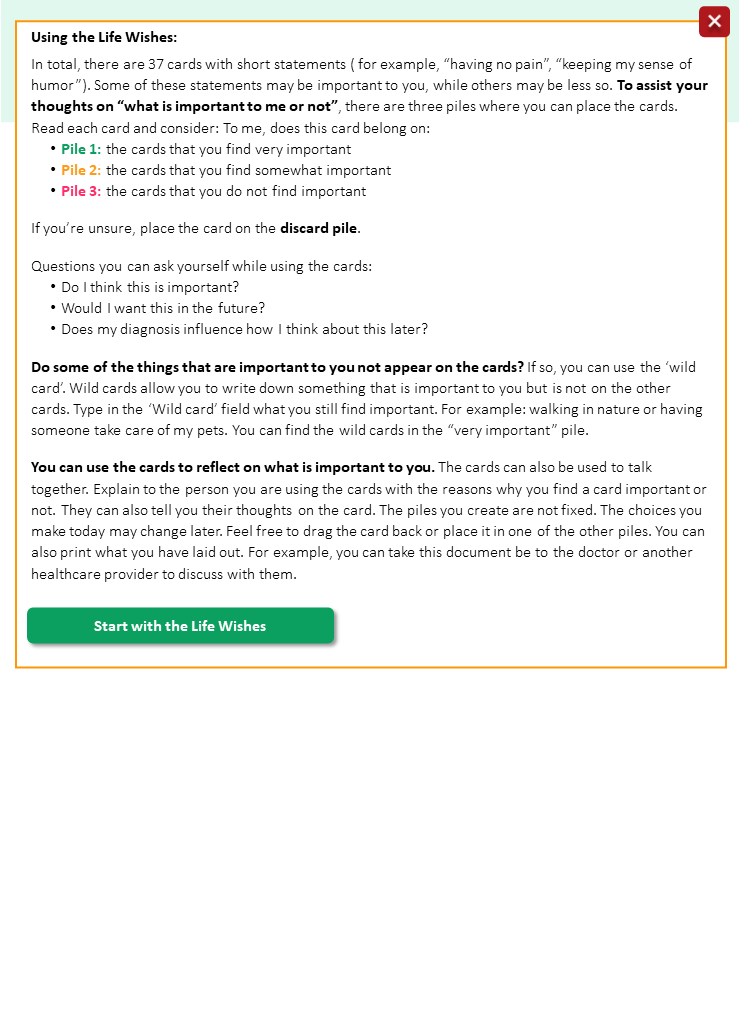
**

**
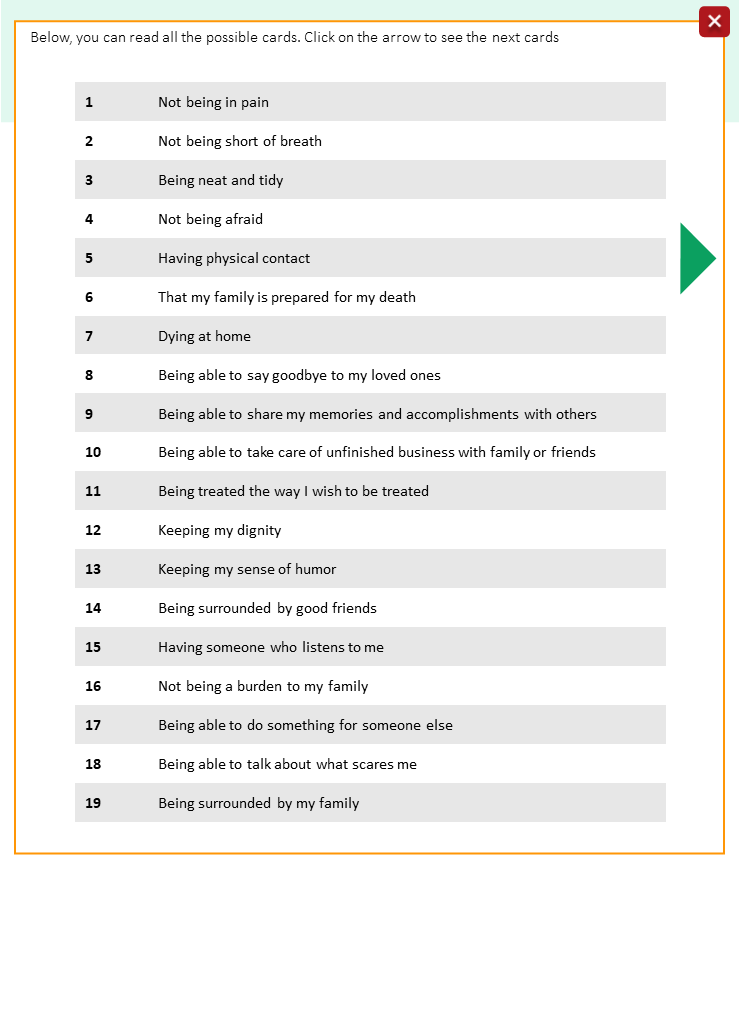
**

**
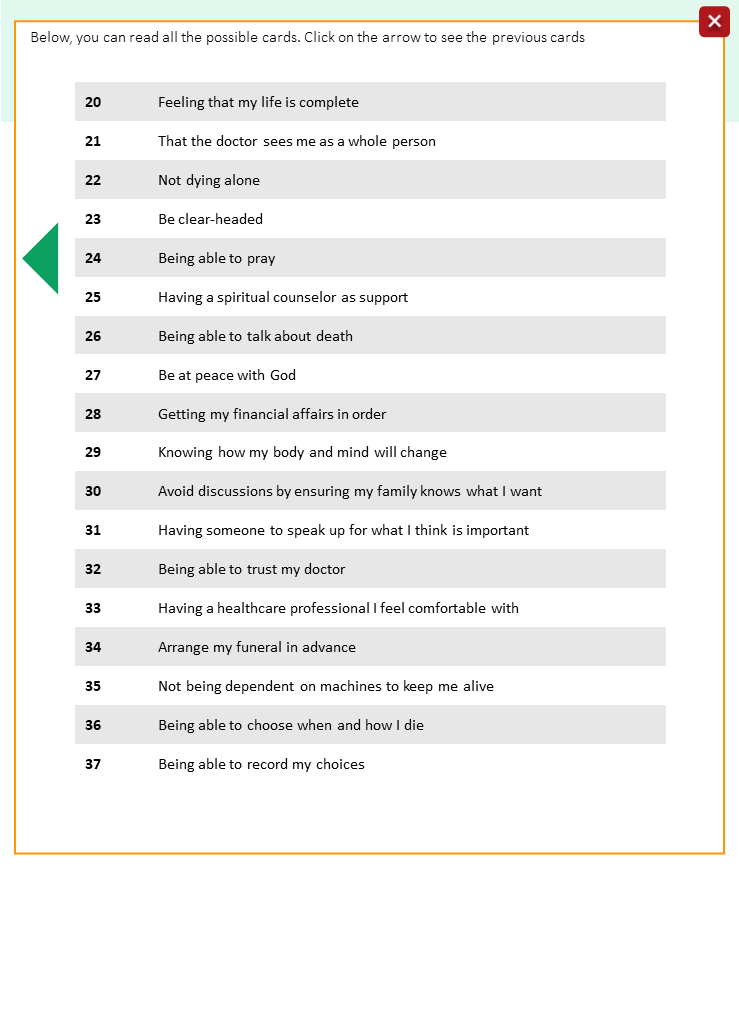
**

**Appendix 3:** Interview topic guide

| **Introduction** |
| --- |
| **Interview questions on the website as a whole on the following topics:**   - Awareness and knowledge of ACP - Reflections and experiences about ACP - Experiences with advance directives - Usability of the ACP support website - Acceptability of the ACP support website - Feasibility of the ACP support website |
| **User experiences with the website and the interactive tools**   - How did you experience using the website? (Discuss different elements of the website, including the Life Wishes and the ‘Thinking Now About Later’ tool) - How did you feel about the interactive tools? Were they useful? Did you experience any difficulties? - How did you feel when using the website, and the two interactive tools? - Did you feel you could apply the tips discussed in the interactive tools? - Would you keep using the tools in the future? |
| **Conclusion** |
